# A Signaling Lipid Associated with Alzheimer’s Disease Promotes Mitochondrial Dysfunction

**DOI:** 10.1038/srep19332

**Published:** 2016-01-13

**Authors:** Michael A. Kennedy, Tia C. Moffat, Kenneth Gable, Suriakarthiga Ganesan, Karolina Niewola-Staszkowska, Anne Johnston, Corey Nislow, Guri Giaever, Linda J. Harris, Robbie Loewith, Vanina Zaremberg, Mary-Ellen Harper, Teresa Dunn, Steffany A. L. Bennett, Kristin Baetz

**Affiliations:** 1 Ottawa Institute of Systems Biology, Department of Biochemistry, Microbiology, and Immunology, University of Ottawa, Ottawa, Ontario, KlH 8M5 Canada; 2 Department of Biochemistry, Uniformed Services University of the Health Sciences, Bethesda, Maryland 20184-4799, USA; 3 Department of Biological Sciences, University of Calgary, AB, T2N 1N4 Canada; 4 Department of Molecular Biology and Swiss National Center for Competence in Research Programme Chemical Biology, University of Geneva, Geneva 1211, Switzerland; 5 Ottawa Research and Development Centre, Agriculture and Agri-Food Canada, Ottawa, Ontario K1A 0C6, Canada; 6 University of British Columbia, Vancouver, British Columbia V6T 1Z3, Canada

## Abstract

Fundamental changes in the composition and distribution of lipids within the brain are believed to contribute to the cognitive decline associated with Alzheimer’s disease (AD). The mechanisms by which these changes in lipid composition affect cellular function and ultimately cognition are not well understood. Although “candidate gene” approaches can provide insight into the effects of dysregulated lipid metabolism they require a preexisting understanding of the molecular targets of individual lipid species. In this report we combine unbiased gene expression profiling with a genome-wide chemogenomic screen to identify the mitochondria as an important downstream target of PC(*O*-16:0/2:0), a neurotoxic lipid species elevated in AD. Further examination revealed that PC(*O*-16:0/2:0) similarly promotes a global increase in ceramide accumulation in human neurons which was associated with mitochondrial-derived reactive oxygen species (ROS) and toxicity. These findings suggest that PC(*O*-16:0/2:0)-dependent mitochondrial dysfunction may be an underlying contributing factor to the ROS production associated with AD.

The current estimate suggests that by 2030 the economic impact of caring for the ~65 million people suffering with Alzheimer’s disease (AD) worldwide will likely exceed 1 trillion dollars annually^1^. This staggering reality underscores the importance of efforts aimed at identifying the molecular mechanisms which contribute to the development of this prevalent disease. While dysregulated lipid metabolism has long been associated with the neurodegenerative events of AD^2^ the relatively recent emergence of comprehensive lipidomic technologies has clarified the nature of these changes identifying aberrant lipid metabolism as a key contributing factor in AD pathology^3^,^4^,^5^,^6^,^7^,^8^,^9^. Converging lipidomic, genetic, and direct biochemical assessments have revealed a particularly compelling link between the accumulation of PC(*O*-16:0/2:0), a platelet activating factor (PAF), and AD pathology mediated through soluble β-amyloid_1-42_ (Aβ_42_)^8^,^9^,^10^. The exact nature of the molecular mechanism(s) by which PC(*O*-16:0/2:0) compromises neuronal function, however, are not completely understood.

By leveraging a systems level approach in the budding yeast *Saccharomyces cerevisiae,* we previously identified phospholipase D (PLD) as an important cellular target involved in buffering against the neurotoxic effects of PC(*O*-16:0/2:0)^11^. This connection between dysregulated PC(*O*-16:0/2:0) metabolism and the documented involvement of PLD in AD pathogenesis and cognitive function prompted further investigation^12^,^13^,^14^,^15^. Interestingly, a subsequent effort to examine the mechanism(s) underlying the protective effects of PLD activity unexpectedly identified an independent role for PC(*O*-16:0/2:0) as a potent inhibitor of Ypk1 phosphorylation by the target of rapamycin complex 2 (TORC2)^16^. Since TORC2-dependent Ypk1 phosphorylation is required for maintaining cellular viability in the presence of PC(*O*-16:0/2:0) our results suggest that downstream targets of Ypk1 may play an important role in mediating the neurotoxic properties of this lipid when its cellular levels are pathologically elevated.

Ypk1 signaling has been implicated in modulating a complex regulatory network involved in coordinating sphingolipid biosynthesis^17^,^18^,^19^,^20^,^21^. The current evidence supports a homeostatic model wherein changes in the relative abundance of complex sphingolipids as well as bioactive sphingolipid precursor metabolites such as ceramide and long chain bases (LCB) act through TORC2-Ypk1 to modulate the activity of enzymes and important regulatory proteins within the sphingolipid biosynthetic pathway^18^. Importantly, inhibition of Ypk1 signaling, as is evident in PC(*O*-16:0/2:0) treated cells, leads to marked impairments in sphingolipid metabolism which ultimately gives rise to the accumulation of ROS as a result of mitochondrial dysfunction and impaired vacuolar acidification^19^. Although dysregulated sphingolipid metabolism, mitochondrial dysfunction, and ROS accumulation are part of AD pathogenesis, it has yet to be determined whether these processes result from a single or converging cascade(s) of metabolic impairments triggered by Aβ_42_ biogenesis^6^,^22^,^23^,^24^,^25^.

Here, we report the findings of an expanded chemogenomic screen and transcriptome profiling approach that provides systems level support for a role of PC(*O*-16:0/2:0) in mediating mitochondrial dysfunction and ROS accumulation in yeast. These findings were validated mechanistically in human neurons wherein we show that ectopically elevating PC(*O*-16:0/2:0) to levels detected in AD temporal cortex *in vivo* and in response to toxic concentrations of Aβ_42_ *in vitro* causes an increase in cellular ceramide abundance, disrupts mitochondrial membrane potential, and stimulates ROS production. Together, these findings provide mechanistic insight into the metabolic cascade triggered by aberrant lipid metabolism at the molecular level. They indicate that the specific disruption in PC(*O*-16:0/2:0) metabolism detected in AD brain mechanistically contributes to mitochondrial dysfunction and ROS production.

## Results

### Chemogenomic signature of PC(*O*-16:0/2:0) identifies a critical role for mitochondrial function

**Figure 1:**
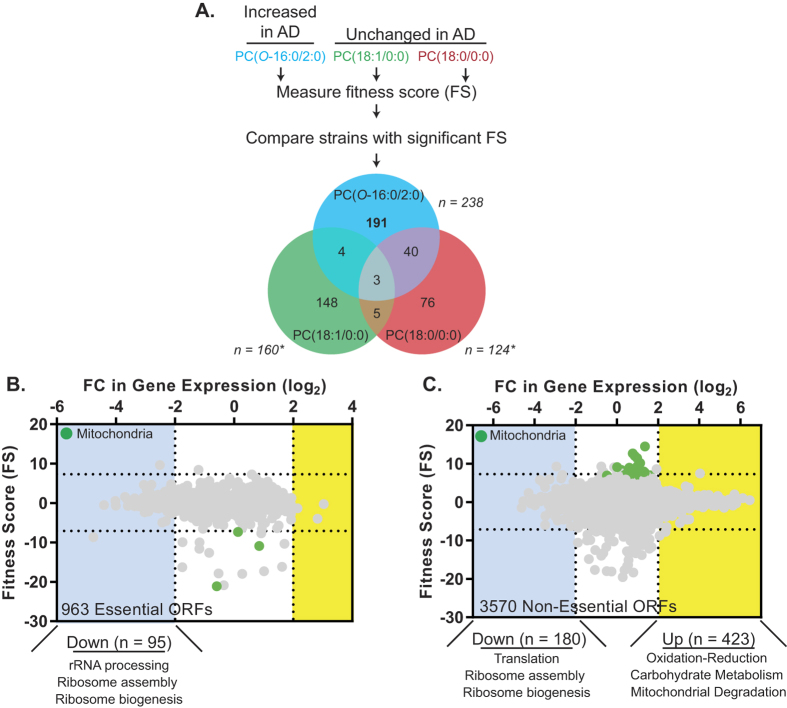
Identification of a mitochondrial role in mediating the cellular response to PC(*O*-16/2:0). (**A**) *Workflow of systems level profiling of the cellular response to PC(O-16:0/2:0).* A *S. cerevisiae* library composed of strains harboring single gene deletions for nearly all essential and non-essential ORFs were grown in the presence of each indicated lipid species. A fitness score (FS) was calculated for each strain following growth with each lipid as described in materials and methods. The total number of ORFs with significant FS is indicated (n) for each lipid. Strains exhibiting significant FS for more than one lipid were removed from subsequent analysis. **(B,C)** *Mitochondrial ORFs are enriched in chemogenomic profile of PC(O-16:0/2:0).* FS for essential (**B**) and non-essential (**C**) ORFs are plotted on the y-axis where FS values >6.7 and FS < −6.7 (horizontal dotted lines) represent an increased or decreased sensitivity respectively. The colored insets (purple and green) identify an enrichment of ORFs with similar cellular localization as classified by gene ontology (GO). No enrichment in cellular localization or biological process was observed for either control lipid. The effects of PC(*O*-16:0/2:0) (40 μM, for 60 min) upon gene expression in wild type yeast (YPH500) are represented on the x-axis with the log_2_-fold change (FC) in the expression of each ORF compared to vehicle treated cells where FC values >2 (yellow) and FC < 2 (blue) represent upregulated and downregulated ORFs respectively. The relative enrichment of GO terms is displayed below the respective graphs. The total number of ORFs included in the final analysis displayed in (**B**) and (**C**) are indicated in the upper right corner.

Previously, we utilized a chemogenomic approach to assess the role of all non-essential open reading frames (ORFs) in mediating PC(*O*-16:0/2:0) toxicity^11^. While this approach demonstrated that PLD regulates PC(*O*-16:0/2:0) toxicity, subsequent follow up studies revealed a distinct, non-overlapping role for the essential target of rapamycin signaling complex 2 (TORC2)^16^. As our initial chemogenomic screen only assessed the involvement of non-essential genes, we sought here to determine whether other essential genes might also mediate cellular responses specific to PC(*O*-16:0/2:0) and thus identify pathways through which this lipid second messenger participates in signaling AD pathology. Using both haploinsufficiency profiling (HIP, essential ORFs) and homozygous profiling (HOP, non-essential ORFs) platforms, we have now assessed the affect of nearly every ORF in the budding yeast genome on the cellular response to PC(*O*-16:0/2:0) (~5900 ORFs in total) (Fig. 1 and Dataset 1). Importantly, this expanded approach permitted the identification of deletion mutants with an increased resistance to PC(*O*-16:0/2:0). To ensure that the observed changes in growth or fitness scores (FS) were specific to PC(*O*-16:0/2:0), we simultaneously assessed FS for two related glycerophosphocholine lipids which have not been implicated in AD (PC(18:1/0:0) and PC(18:0/0:0)) (Fig. 1A). A similar number of ORFs were found to confer a significant FS with each individual lipid species (Fig. 1A see reported n value for each lipid). The majority of the ORFs had a significant FS with only one lipid indicating distinct cellular targets (and downstream pathways) exist for each lipid species (Fig. 1A)^11^. Of the 191 mutants which exhibited significant FS specific to PC(*O*-16:0/2:0) (HIP – 23 and HOP – 168) there was a significant enrichment (Holm-Bonferonni correction) in ORFs involved in mitochondrial translation (GO:0032543, 22 ORFs, p-value:5.8e-7) and mitochondria organization (GO:0007005, 35 ORFs, p-value:1.9e-6) (Fig. 1B,C and Dataset 1). These findings are in contrast to the two control lipid species, PC(18:1/0:0) (160 total ORFs) or PC(18:0/0:0) (124 total ORFs), where no enrichment for these or other biological processes were observed despite a similar number of ORFs identified. Therefore, since our chemogenomic profiling identified a critical role for the mitochondria we sought to further examine the role of mitochondrial function in mediating the effects of PC(*O*-16:0/2:0) upon cellular growth.

### PC(*O*-16:0/2:0) induces expression of oxidative stress response genes

To provide additional insight into the nature of this mitochondrial response, we assessed transcriptional changes elicited by PC(*O*-16:0/2:0) (Fig. 1B,C and GSE40814). We observed significant (greater than 2-fold) reprogramming of the transcriptome with a total of 275 ORFs down regulated (95 essential, 180 non-essential) and 426 ORFs upregulated (3 essential, 423 non-essential) (Fig. 1B,C and Dataset 1). Transcripts involved in ribosome assembly and ribosome biogenesis were primarily downregulated in response to PC(*O*-16:0/2:0) whereas upregulated transcripts were enriched for ORFs involved in regulating cellular oxidative stress (GO:0006979, 22 ORFs, p-value:6.3e-4) and mitochondrial degradation (GO:0000422, 10 ORFs, p-value:1.3e-2) (Holm-Bonferroni correction). Taken together, these comprehensive unbiased screens identify a critical role for the mitochondria in buffering the toxicity of PC(*O*-16:0/2:0).

### PC(*O*-16:0/2:0) elicits ROS production

**Figure 2:**
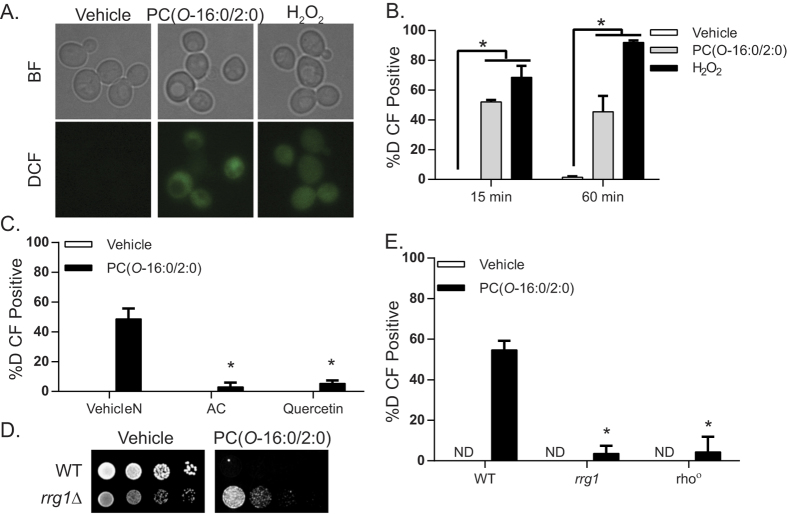
Mitochondria are the major source of ROS production in PC(*O*-16:0/2:0) treated cells. (**A**) *H2-DCFDA fluorescence is increased in PC(O-16:0/2:0) treated cells.* Wild type (BY4741) cells were grown to mid log in YPD and subsequently treated with Vehicle (0.2% Ethanol), PC(*O*-16:0/2:0) (20 μM) or H_2_O_2_ (0.8 mM) for t = 15 or 60 min prior to labelling with H_2_-DCFDA (10 μM). Representative brightfield (BF) and H2-DCFDA (DCF) images from t = 15 min are shown. (**B**) Quantifications of H2-DCFDA positive cells at the indicated time points from at least three independent experiments where a minimum of 150 cells were counted. Error bars = SD, (*p < 0.01, Kruskal-Wallis test). **(C)** *Antioxidants reduce H2-DCFDA fluorescence.* Wild type (BY4741) cells were grown to mid log in YPD with or without n-acetyl cysteine (NAC 20 mM, pH 7.5) or quercetin (300 μM) and subsequently treated with PC(*O*-16:0/2:0) (20 μM, 15 min) and labeled with H2-DCFDA (10 μM) and positive cells were quantified. A minimum of 150 cells from three independent experiments were counted. Error bars = SEM, (*p < 0.01, Kruskal-Wallis test). **(D)** *Inhibition of ROS production reduces PC(O-16:0/2:0) toxicity.* Sensitivity of wild type (WT, BY4741) and *rrg1*Δ (YKB3911) strains were assessed by examining growth on plates containing vehicle (Ethanol, EtOH) or PC(*O*-16:0/2:0) (7 μg/mL or 13.4 μM) at 30 °C for 2 days. Representative image from 3 experiments is shown. **(E)** *Mitochondrial respiration is required for ROS production in PC(O-16:0/2:0) treated cells.* Wild type (BY4741), *rrg1*Δ (YKB3911), *rho°* (YKB3925) cells were grown to mid log, treated with PC(*O*-16:0/2:0) (20 μM, 15 min) and labeled with H2-DCFDA (10 μM). A minimum of 150 cells from three independent experiments were counted. Error bars = SEM, (*p < 0.05, Kruskal-Wallis test). ND – not detected.

To validate the systems level association between the effect of PC(*O*-16:0/2:0) on mitochondrial function and levels of oxidative stress (Fig. 1B,C and Dataset 1), we first asked if the treatment elicited ROS production by assessing H2-DCFDA fluorescence (Fig. 2A,B). A significant increase in the percentage of cells exhibiting increased ROS production was observed as early as 15 min after exposure to PC(*O*-16:0/2:0) but not control lipids (Fig. 2A,B and Supplemental Fig. 1A). An increase in mitochondrial fragmentation was also detected following treatment further suggesting that PC(*O*-16:0/2:0) is indeed affecting mitochondrial function (Supplemental Fig. 1). Pretreating cells with the antioxidants *N*-acetylcysteine or quercetin significantly reduced the percentage of H2-DCFDA-positive cells with accumulated ROS to nearly untreated levels and reduced PC(*O*-16:0/2:0) toxicity (Fig. 2C). To verify mitochondrial-dependent ROS production, we compared response of a wild type strain with mutants defective in mitochondrial respiration. First we examined ROS production in the *rrg1*Δ deletion mutant, a single ORF required for respiration^26^ that also displayed increased resistance to PC(*O*-16:0/2:0) in both the chemogenomic screen and confirmatory growth assay (Dataset 1 and Fig. 2D). In addition, we examined ROS production in a strain devoid of mitochondrial DNA (*rho°*) (Fig. 2E)^27^. The respiration deficient properties of both strains were confirmed by the absence of growth on non-fermentable carbon sources (Supplemental Fig. 1). Notably we found that ROS production was equally and significantly reduced (to essentially untreated wild type levels) in both strain backgrounds suggesting that mitochondria are the major source of ROS production following PC(*O*-16:0/2:0) treatment (Fig. 2E).

### PC(*O*-16:0/2:0)-induced LCB(P) and ceramide accumulation signal ROS formation through an inhibition of TORC2-Ypk1 signaling

**Figure 3:**
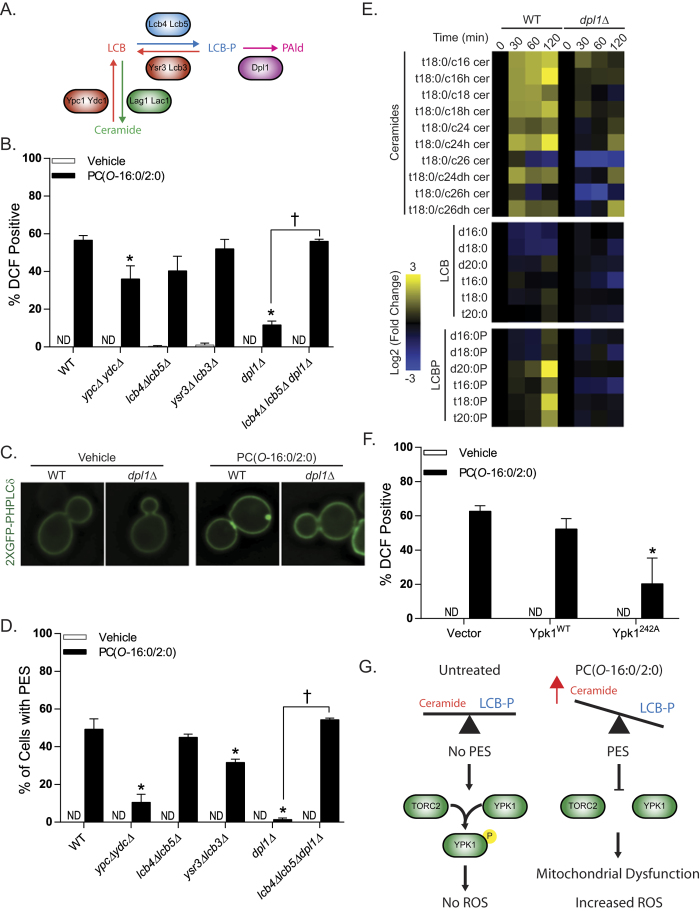
PC(*O*-16:0/2:0)-dependent inhibition of TORC2-Ypk1 signaling to the mitochondria contributes to ROS production. (**A**) *Simplified representation of sphingolipid metabolic pathway in yeast.* **(B)** *Identification of ceramide metabolism mutants involved in ROS generation.* Wild type (WT, BY4741), *ypc1*Δ *ydc1*Δ (YKB3271), *lcb4*Δ *lcb5*Δ (YKB3273), *ysr3*Δ *lcb3*Δ (YKB3305), *dpl1*Δ (YKB3306) and *lcb4*Δ *lcb5*Δ *dpl1*Δ (YKB3927) strains were grown to mid-log, treated with PC(*O*-16:0/2:0) (20 μM, 15 min) and labeled with H2-DCFDA (10 μM). **(C)** and **(D)** *ROS-deficient ceramide mutants do not exhibit PES formation.* The subcellular localization of PtdIns(4,5)P_2_ pools were assessed in the indicated strains expressing a GFP-tagged PtdIns(4,5)P_2_ probe as previously done (pRS416-GFP2xPH^PLCδ^)^16^. Redistribution of the probe was not detected (ND) in any of the untreated strain backgrounds as shown in the representative images from Wild type (WT, BY4741) and *dpl1*Δ (YKB3306) backgrounds. **(E)** *PC(O-16:0/2:0) treatment disrupts ceramide metabolism*. Wild type (WT, BY4741) and *dpl1*Δ strains were treated with vehicle or PC(*O*-16:0/2:0) (20 μM) for the indicated times (min). Lipids were extracted and levels were quantified and expressed as a log2 fold change of PC(*O*-16:0/2:0) treated from vehicle treated isogenic control. LCB, long chain base; LCBP, long chain base phosphate **(F)***The hyperactive allele of the TORC2 target Ypk1 prevents ROS production*. Wild type (BY4741) cells expressing empty vector (Vector, pRS416), wild type Ypk1 (Ypk1^WT^) or hyperactive Ypk1^D242A^ (Ypk1^D242A^) were grown to mid log, treated with PC(*O*-16:0/2:0) (20 μM, 15 min) and labeled with H2-DCFDA (10 μM). Quantification for all experiments represents the average percentage of positive cells from at least three independent experiments where a minimum of 150 cells were counted. Error bars = SEM. (*p < 0.05 Kruskal-Wallis). ND – not detected. **(G)** *Model of potential mechanism by which PC(O-16:0/2:0) inhibits TORC2/Ypk1 signaling via the ceramide/LCBP rheostat.* Elevated concentrations of PC(*O*-16:0/2:0) promote an increase in ceramide levels which are associated with PES formation and inhibition TORC2-dependent Ypk1 phosphorylation leading to impaired mitochondrial function and increased production of ROS.

Previously we reported that elevated concentrations of PC(*O*-16:0/2:0) promote an accumulation of long chain bases (LCBs), their phosphorylated derivatives (LCB-Ps) and ceramide species, three upstream metabolites within the sphingolipid biosynthetic pathway (Fig. 3A)^16^. Therefore, we next sought to determine whether the accumulation of LCB(P)s and/or ceramides contribute to ROS production upon exposure to PC(*O*-16:0/2:0) using a complement of deletion mutants targeting each of the enzymatic activities within this pathway (Fig. 3A). Interestingly, deletion of either ceramidase activity (*ypc1*Δ *ydc1*Δ) or LCB-P lyase (*dpl1*Δ) activity resulted in a significant reduction in ROS production (Fig. 3B). Furthermore, inhibition of ROS production in the *dpl1*Δ strain required the formation of LCB-P as deletion of the LCB kinases *lcb4*Δ *lcb5*Δ in a *dpl1*Δ background resulted in wild type levels of ROS production (Fig. 3B). Together these findings suggest that increased LCBP levels can significantly reduce ROS production in response to PC(*O*-16:0/2:0).

How might an increase in LCBP levels regulate ROS production? Previously we demonstrated that PC(*O*-16:0/2:0)-induced ceramide accumulation led to an enrichment of phosphatidyl-inositol-4,5-phosphate (PtdIns(4,5)P_2_) to distinct locations in the plasma membrane which we termed PtdIns(4,5)P_2_ enriched structures (PES)^16^. The formation of these structures is associated with an inhibition of target of rapamycin complex 2 (TORC2)-Ypk1 signaling axis. Interestingly, a recent report by the Powers group has identified a critical role for TORC2-Ypk1 in regulating ROS production from the mitochondria^19^. Therefore, we initially assessed PES formation as a proxy of TORC2-Ypk1 inhibition in our complement of LCB(P)-ceramide mutants (Fig. 3C,D). In agreement with our ROS findings both the ceramidase and LCB-P lyase strains exhibited significant reductions in PES formation whereas the *lcb4*Δ *lcb5*Δ *dpl1*Δ triple mutant did not (Fig. 3C,D) suggesting that elevated LCBP levels prevent the inhibitory effects of PC(*O*-16:0/2:0) upon Ypk1 activity by preventing the deleterious effects of this lipid upon sphingolipid metabolism. To investigate this possibility directly we measured the relative changes in the abundance of sphingolipid metabolites in wild type and *dpl1*Δ strains treated with PC(*O*-16:0/2:0) (Fig. 3E and Dataset 2). In agreement with our previous findings, we observed that PC(*O*-16:0/2:0) induced an increase in the majority of ceramide species within the sphingolipid metabolic pathway in wild type cells treated with PC(*O*-16:0/2:0) (Fig. 3E). However, the accumulation of ceramide and LCBP lipid species was greatly attenuated in a *dpl1*Δ mutant further suggesting that PC(*O*-16:0/2:0)-dependent ROS production is dependent upon altered sphingolipid metabolism and impaired TORC2-Ypk1 signaling (Fig. 3E). The role of Ypk1 activity in mediating PC(*O*-16:0/2:0)–dependent ROS production was further confirmed by the significant reduction in ROS following overexpression of a hyperactive allele of Ypk1 (242A^28^) in wild type cells (Fig. 3F).

### PC(*O*-16:0/2:0) disrupts ceramide metabolism and promotes ROS production in cultured human neurons

**Figure 4:**
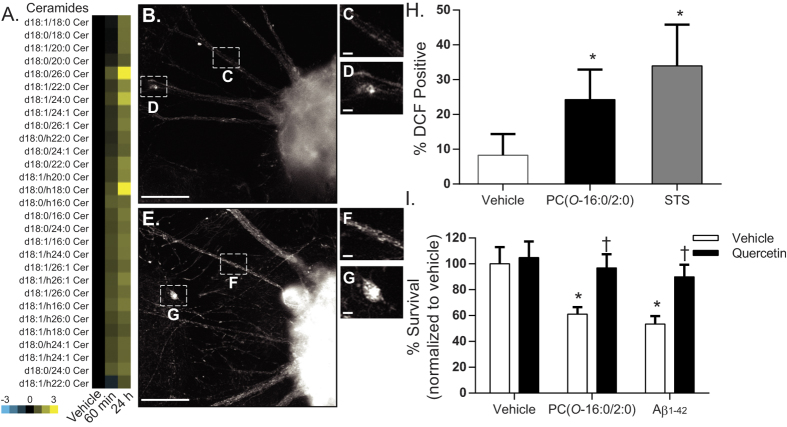
Inhibition of ROS production prevents PC(*O*-16:0/2:0) and Aβ_42_ neurotoxicity. (**A**) *PC(O-16:0/2:0) promotes increased ceramide abundance in cultured human neurons.* hNT cells were treated with vehicle or PC(*O*-16:0/2:0) (1 μM). At 60 min or 24 h lipids were extracted and relative abundance of ceramide species determined. Heat map represents the normalized log_2_ fold change in ceramide abundance of PC(*O*-16:0/2:0) in compared to control from three independent cultures. (see Dataset 2 for normalized data). (**B**) *PC(O-16:0/2:0) affects mitochondrial membrane potential in cultured neurons.* hNT cells were treated for 24 h with either vehicle (**B–D**) or 1 μM PC(*O*-16:0/2:0) **(E–G)** were labelled with the mitochondrial dye MTR. Punctate mitochondrial MTR staining is seen throughout the neuritic extensions and the neuronal cell bodies. PC(*O*-16:0/2:0) induced an increase in overall neuronal MTR fluorescence intensity, indicative of an increase in either ΔΨ_m_ or ROS levels. Scale bars, 50 μm. Inset, 10 μM. (**H**) *ROS levels are increased in PC(O-16:0/2:0) treated neurons.* The % of ROS-positive hNTs was quantified using the H2-DCFDA assay. A 24 h treatment of 1 μM PC(*O*-16:0/2:0) induced a 3-fold increase in hNT ROS levels. STS was used as a positive control (*p < 0.001, ANOVA, *post-hoc* Dunnett’s t test). **(I)** *Antioxidants reduce PC(O-16:0/2:0) and A*β_*42*_ *toxicity in neuronal cultures*. hNT cells pretreated with quercetin (3.3 μM) were subsequently treated with vehicle (0.1% v/v Ethanol/0.1% v/v DMSO), PC(*O*-16:0/2:0) (1 μM) or Aβ_42_ (25 μM) for 24 h. Cell viability was assessed using the TUNEL assay. Data are standardized to vehicle control and expressed as mean ± SEM (n = 27-45 fields per data point from 5 separate cultures). * indicates a significant decrease in % survival of cells relative to controls cultures treated with 0.1% Ethanol / 1% DMSO (Vehicle control) p < 0.01, ANOVA, *post-hoc* Dunnett’s t test. †, indicates a significant increase in % survival of cells treated with quercetin, p < 0.05, ANOVA, *post-hoc* Dunnett’s t test compared to relevant treatment.

To determine whether the effects of PC(*O*-16:0/2:0) in *S. cerevisiae* model the responses seen in human neurons, we next examined the affect of PC(*O*-16:0/2:0) in differentiated hNT2 cells, a well characterized model that recapitulates many properties of human neuronal cells such as neurotransmitter expression and the formation of synapses^29^,^30^,^31^. We have previously demonstrated that increasing intracellular concentrations of PC(*O*-16:0/2:0) by either the addition of exogenous lipid or Aβ1-42 oligomers is sufficient to initiate caspase activation and cellular death in this background. However, the affect of elevated intracellular concentrations of PC(*O*-16:0/2:0) upon sphingolipid metabolism and mitochondrial function has not been investigated. In agreement with our findings in yeast^16^, we report that PC(*O*-16:0/2:0) (1 μM) elicits an increase in global ceramide abundance as early as 60 min which is sustained for up to 24 h (Fig. 4A, Dataset 3). In addition, treatment with PC(*O*-16:0/2:0) also resulted in an increase in mitotracker red (MTR) fluorescence in comparison to vehicle-treated cells at 24 h suggesting a disruption in mitochondrial membrane polarization (ΔΨ_m_) or ROS production (Fig. 4B–G)^32^,^33^. To distinguish between these possibilities, ROS production and mitochondrial membrane potential were directly measured using H2-DCFDA and TMRE (Fig. 4H and Supplemental Fig. 2). As observed in *S. cerevisiae,* a significant increase in ROS production was evident following exposure to PC(*O*-16:0/2:0) whereas no change in mitochondrial membrane potential was detected (Fig. 4H and Supplemental Fig. 3). Finally, pre-treating cultured neurons with the anti-oxidant quercetin was sufficient to inhibit the toxic effects of both PC(*O*-16:0/2:0) and Aβ_42_ as evident in the reduced percentage of TUNEL positive cells (Fig. 4I). Together our results suggest that the neurotoxicity associated with elevated intraneuronal concentrations of PC(*O*-16:0/2:0) is in part dependent upon changes in mitochondrial dysfunction and ROS production.

## Discussion

A number of independent studies aimed at profiling the AD “neurolipidome” have identified significant changes in the relative abundance of a number of lipid species but the role of these changes in disease pathology are not well understood (see^34^ for an overview of these studies). In this report, we utilized a combination of genome wide chemogenomic and transcriptome profiling approaches to identify a critical role for mitochondrial-derived ROS in mediating the cytotoxic effects of a platelet activating factor lipid species PC(*O*-16:0/2:0) which is elevated in AD. We also provide evidence that compounds with antioxidant properties that reduce ROS can protect cultured human neurons from the toxic effects of PC(*O*-16:0/2:0). Importantly, these findings suggest that the mitochondrial dysfunction and increased ROS present in AD may arise as a result of dysregulated PC(*O*-16:0/2:0) metabolism and approaches which mitigate the effects of this lipid upon could have significant therapeutic benefit in this neurodegenerative context.

Expanding upon our previous work with PC(*O*-16:0/2:0)^11^,^16^ our current results now includes the contribution of all essential and non essential ORFs to the fitness of cells treated with this bioactive lipid (Fig. 1 and Dataset 1). In contrast to our previous study this approach permitted the identification of the mitochondria as an important cellular target because we were also able to identify ORFs which suppress the growth inhibitory effects of PC(*O*-16:0/2:0) (Fig. 1 and Dataset 1). This finding was intriguing because the etiology of mitochondrial dysfunction in AD is not well understood. Complementing our chemogenomic screen with the first reported examination of the transcriptional response to PC(*O*-16:0/2:0) we determined that elevated concentrations of PC(*O*-16:0/2:0) contribute to the upregulation of transcripts involved in mediating the cellular response to oxidative stress and mitochondrial degradation (Fig. 1 and Dataset 1).

The combined evidence presented here are consistent with previous findings implicating PC(*O*-16:0/2:0) as an inducer of ROS in a variety of non-neuronal cell types. However, little evidence currently supports a direct role for PC(*O*-16:0/2:0) in generating ROS in neuronal cells by disrupting mitochondrial function. Furthermore, the PC(*O*-16:0/2:0)-dependent stimulation of ROS in non-neuronal cells is primarily thought to occur in response to activation of a signaling cascade involving the PAF receptor which is notably absent in yeast and select neuronal cell populations^35^,^36^,^37^,^38^. Together these data suggest PC(*O*-16:0/2:0)-dependent increases in ROS occurs through a different mechanism in yeast and neuronal cells which is dependent upon mitochondrial dysfunction.

A recent report by Niles *et al.* determined that mitochondrial function and production of ROS are regulated by the TORC2-Ypk1 signaling axis, a signaling pathway which we have previously shown to be inhibited by PC(*O*-16:0/2:0)^16^,^19^. Our current data suggests that the inhibition of Ypk1 activation by PC(*O*-16:0/2:0) is in part responsible for the observed mitochondrial dysfunction and increased ROS (Fig. 3). The mechanism by which Ypk1 affects mitochondrial function to regulate ROS production is incompletely understood but does involve the hyperactivation of Tpk3, a catalytic subunit of PKA^19^. Interestingly, hyperactivation of PKA was previously shown to increase ROS production in part through downregulating transcripts of the mitochondrial electron transport system (ETS)^39^. However, our data do not support a role for increased PKA activity in mediating mitochondrial dysfunction and ROS production in PC(*O*-16:0/2:0) treated cells as single deletions of PKA catalytic subunits (*tpk1*Δ, *tpk2*Δ or *tpk3*Δ) or the phosphodiesterase (*pde2*Δ) did not affect cellular fitness as might be expected when grown in the presence of PC(*O*-16:0/2:0) (Dataset 1). In addition, PKA-responsive transcripts of the ETS were not significantly impacted by the addition of PC(*O*-16:0/2:0). The discrepancy between these findings can likely be attributed to the methods by which Ypk1 activity was inhibited. While Niles *et al.* targeted a genetically modified version of Ypk1 with a small molecule our previously published data suggests that PC(*O*-16:0/2:0) inhibits TORC2-dependent activation of Ypk1 indirectly through changes in sphingolipid metabolism.

Current evidence suggests that depletion of sphingolipid metabolites such as ceramide and long chain bases (LCBs) will stimulate TORC2, an essential signaling complex, to phosphorylate and activate Ypk1 which in turn promotes the activity of a number of enzymes and regulatory proteins responsible for the production of these sphingolipids metabolites. This elegant mechanism ensures that enzyme activity is coordinated with the relative abundance of sphingolipid precursor molecules such as ceramide and long chain bases to enable cellular growth. Although the exact nature of the molecular signals which regulate this signaling loop have remained largely enigmatic it is clear that a reduced availability of sphinoglipid metabolites themselves are able to increase Ypk1 dependent activation by causing a redistribution of PtdIns(4,5)P_2_ and respective TORC2 adaptor proteins through poorly defined mechanisms. Importantly, we have previously reported complementary results following treatment with PC(*O*-16:0/2:0) which suggest increased availability of sphingolipid metabolites have the opposite effect on TORC2-dependent Ypk1 activation. In particular, we have found that PC(*O*-16:0/2:0) causes an increased abundance of ceramide, LCBs and phosphorylated derivatives (LCBPs) is associated with a dramatic redistribution of PtdIns(4,5)P_2_ and inhibition of Ypk1 phosphorylation. Our current finding that a deletion of either ceramidase (*ypc1*Δ *ydc1*Δ) or LCB lysase (*dpl1*Δ) activity significantly inhibits PtdIns(4,5)P_2_ redistribution and ROS production is indicative of a role for ceramide and LCB lipid species in regulating mitochondrial function through inhibition of TORC2-Ypk1 signaling (Fig. 3E).

A primary goal of this work was to identify cellular targets of PC(*O*-16:0/2:0) which could be used to inform our understanding of the role this lipid plays in AD. Importantly, mitochondrial dysfunction and ROS are implicated in the development and progression of AD by multiple lines of evidence (reviewed in^24^,^25^). Our current findings suggest that an intraneuronal accumulation of PC(*O*-16:0/2:0) alone is sufficient to disrupt mitochondrial function and increase ROS in cultured human neurons (Fig. 4). Importantly, our observations are in agreement with a previous finding and suggest that the increase in ROS is largely responsible for the toxic properties of PC(*O*-16:0/2:0). However, TORC2-Ypk1 signaling is known to regulate a diverse number of cellular processes including recent work demonstrating an essential role for this pathway in regulating autophagic flux^40^. Future studies will be needed to determine the relative contribution of these pathways to the overall neurotoxic properties of this lipid. Additionally, since the deleterious effects of PC(*O*-16:0/2:0) accumulation are attributed to significant accumulation of ceramide further work will be needed to determine if directly modulating this signaling pathway can provide neuroprotective effects. In summary, our present study provides compelling evidence linking dysregulated PC(*O*-16:0/2:0) metabolism with mitochondrial dysfunction, ROS and neurotoxicity.

## Materials and Methods

### Yeast strains and plasmids

Yeast strains used in this study are listed in Supplemental Table 1. Deletion strains were made using standard PCR-mediated techniques as previously described^41^. Wild type Ypk1 (pAH126) and mutant Ypk1^D242A^ 20 were subcloned into the yeast expression vector pRS416^42^. The mitochondrial deficient strain *rho°* (YKB3925) was generated by isolating petite, non-respiring colonies from the BY4741 parental strain treated with ethidium bromide as has been previously described^27^.

### Chemogenomic profiling

The yeast chemical screen and fitness score (FS) calculations were performed essentially as previously described^43^. Significant genetic interactions with the lipids were assigned to ORFs identified as outliers within the data set using the ROUT method^44^.

### Transcriptome Microarray

Single dye microarray experiments and analysis were performed with custom *S. cerevisiae* microarray 8 × 15 K slides (Agilent) as previously described^45^ with the following modifications. Total RNA was isolated from wild type (YPH500) cells treated with vehicle (Ethanol) or PC(*O*-16:0/2:0) (40 μM) for 1 hour. Four independent biological replicates were performed for each condition. Slides were scanned with a resolution of 5 μM and the PMT gains were between 280 and 380 according to the strength of the individual array. The normalization factor was determined so that the average intensity of each array was 1900 (1900/mean of F532 median = normalization factor). Raw data can be accessed at NCBI GEO, accession no. GSE40814.

### Dot assays

Cells were grown in YPD at 30 °C to mid-log phase and resuspended to an OD_600_ of 0.1. Dot assays were performed by spotting 4 μL of ten-fold serial dilutions (OD_600_ = 0.1, 0.01, 0.001, 0.0001) onto YPD plates containing the specified concentrations of Ethanol (vehicle) or PC(*O*-16:0/2:0).

### Microscopy

For all microscopy experiments involving *S. cerevisiae*: overnight cultures grown at 30 °C were re-suspended at a final OD_600_ of 0.2 in specified growth medium and allowed to reach mid-log phase. Cells were incubated with H2-DCFDA (10 μM) and washed in complete media prior to treating with PC(*O*-16:0/2:0) (20 μM). Live cell imaging was performed by briefly centrifuging the cells (800 g for 2 min), followed by resuspending in a minimal volume of growth media, spotting onto glass slides and coverslipping prior to imaging. All images were acquired using a Leica DMI 6000 florescent microscope (Leica Microsystems GmbH, Wetzler Germany), equipped with a Sutter DG4 light source (Sutter Instruments, California, USA), Ludl emission filter wheel with Chroma band pass emission filters (Ludl Electronic Products Ltd., NY, USA) and Hamamatsu Orca AG camera (Hamamatsu Photonics, Herrsching am Ammersee, Germany). Images were acquired at 0.2 μM steps using a 63× oil-immersion objective with a 1.4 numerical aperture. Analysis of H2-DCFDA fluorescence was performed using Velocity Software V4 (Perkin Elmer). For analysis of mitochondria morphology, wild type (BY4741) cells were transformed with were transformed with plasmids carrying gene fusions (Supplemental Table 1) that would target green fluorescent protein (GFP) to different mitochondrial compartments. Expression of GFP fused to the NH2-terminal region of the matrixprotein citrate synthase (CS1), the COOH-end of the inner membrane protease subunit Yta1, or the NH2 terminus of the outer membrane protein Tom6 was driven by the GAL1/10 promoter 1. Cells were grown overnight in defined media containing 2% galactose at 30 °C with shaking (200 rpm). Cultures were diluted in the same medium and grown to mid-log phase. An aliquot of this culture (0.5 A600 units of cells) was treated with 20 μM PC(*O*-16:0/2:0) (Avanti Polar Lipids, Inc.) or incubated with vehicle (0.1% ethanol, untreated control) for 1 hr at 30 °C. Cells were then collected for live imaging analysis, concentrated and placed on slabs of solid medium as previously described2. Coverslips were sealed and cells were imaged using a Zeiss Axiovert 200 M inverted epifluorescence microscope equipped with Axio Cam MRm camera, fitted with EC Plan Neofluar 100×/1.3 Oil M27 objective lens. Images were captured with Axiovision Rel. 4.7 software. The GFP signal was visualized using 470/40 nm bandpass excitation filter, a 495 nm dichromatic mirror and a 525/50 nm bandpass emission filter. Z-stacks were acquired (0.2 μm) and micrographs were deconvolved using the nearest neighbour algorithm. Image J3 and Adobe Photoshop 7.0 were used for image analysis and preparation of figures. GraphPad Prism 5.01 software was used for statistical analysis of data and preparation of figures.

### Human Neuronal Culture and treatment

NT2 cells, originally obtained from Stratagene, were cultured in 10 cm tissue culture plates using Dulbecco’s Modified Eagle Medium:Nutrient Mixture F-12 (DMEM/F12) supplemented with 10% fetal bovine serum (FBS), 1% penicillin/streptomycin, 2 mM L-glutamine (complete media). Cells were passaged every 2–3 days. Terminal differentiation of NT2 cells into human hNT neurons was performed as described in^46^. Terminally differentiated hNT cultures were obtained following 4 weeks of differentiation^46^ and 3 weeks of mitotic inhibition using cytosine β-D-arabinofuranoside (ara-C, 1 μM, Sigma) and 5-fluoro-deoxyuridine (FUdR, 10 μM, Sigma). hNTs were treated with either Ethanol (0.1%; vehicle), PC(*O*-16:0/2:0) (1 μM, BioMol), staurosporine (STS, 1 μM), or Aβ_1-42_, (25 μM). Where indicated cultures were pretreated for 15 min with quercetin (3.3 μM; Dr. John Thor Arnason). Aβ1-42 was prepared as soluble oligomers as previously described^47^. Compounds were diluted serum-free treatment media consisting of DMEM/F12 supplemented with 1% penicillin/streptomycin, 2 mM L-glutamine, and 0.025% bovine serum albumin (BSA, BioShop). Cell survival was assesed by Terminal deoxynucleotidyl transferase deoxyuridine triphosphate nick end labeling (TUNEL, Roche) as we have described^8^. Cells were imaged using a Leica DMRXA2 epifluorescent microscope equipped with a Hamamatsu ORCA-ER digital camera and OpenLab 3.17 software (Improvision).

### ROS Imaging in Human Neurons

ΔΨ_m_/ROS production was measured using a modified protocol from the manufacturer’s instructions for mitochondrial staining with MitoTracker Red CMXRos (MTR, Molecular Probes). Cells were cultured in tissue culture dishes containing glass coverslips coated with laminin/poly-D-lysine. Following treatments, cells were washed in 10 mM phosphate-buffered saline (PBS). MTR reagent was reconstituted in dimethyl sulfoxide (DMSO) and diluted in treatment media to give a 200 nM final concentration. Cells were incubated in the diluted MTR for 15 min at 37 °C and 5% CO_2_, and then washed with PBS and fixed for 20 min in 3.7% paraformaldehyde diluted in PBS. Cells were mounted in Vectashield (Vector Laboratories) and analyzed with a Leica DMRXA2 epifluorescent microscope equipped with an ORCA-ER digital camera (Hamamatsu) and with OpenLab 3.17 software (Improvision). The presence of ROS was measured using a modified protocol from the manufacturer’s instructions for the 5-(and-6)-carboxy-2′,7′-dichlorodihydrofluorescein diacetate assay (H2-DCFDA, Molecular Probes). Cells were cultured in 96-well black plates with clear bottoms. Following treatments, cells were washed with PBS. H2-DCFDA reagent was reconstituted in DMSO and diluted in PBS to give a 100 μM final concentration. Cells were incubated in the diluted H2-DCFDA (100 μL per well) for 30 min at 37 °C and 5% CO_2_, and then washed with PBS. Cells were immediately analyzed using a Leica DMIL inverted epifluorescent microscope equipped with a Qimaging QICAM fast 1394 digital camera (Quorum Technologies). Phase contrast and fluorescent photos were taken of ten random fields per treatment in duplicate experiments, with only one random field taken per well due to the photosensitivity of the H2-DCFDA reagent observed under fluorescent wavelengths.

Mitochondrial membrane potential was assessed using tetramethylrhodamine ethyl ester (TMRE) as we have described^48^ with the exception that a lower concentration of TMRE was used to ensure cells were not analyzed in quench mode. hNT neurons were cultured in 96 well black plates with clear bottoms. Cells were incubated for 24 h in treatment media consisting of phenol red -free DMEM/F12 supplemented with 1% penicillin/streptomycin, 2 mM L-glutamine and (a) 10% FBS (control), (b) serum-free media containing 0.1% EtOH and 0.025% BSA (vehicle control) or (c) serum-free media 1 μM PC(*O*-16:0/2:0) and 0.025% BSA (PAF treatment). Following a 24 h incubation at 37 °C, 50 nM TMRE was added for 10 min and fluorescence read (excitation = 544 nm, emission = 590 nm). After 10 min of recording, 11 uM FCCP was added to each culture and fluorescence assessed for an additional 10 min. At the end of the treatments, cells were lysed and protein content assessed using Bio-Rad DC protein assay according to manufacturer’s protocol. Data are presented as the change in fluorescence intensity averaged over each 10 min recording period normalized per ug of protein average relative to untreated controls. FCCP was used as a positive control to induce proton leak and decrease mitochondrial membrane potential.

### LC/MS sample preparation and analysis

#### Saccharomyces cerevisae

Cells at 0.6 OD600 were treated with 20 μM PAF or ethanol as a control. At T = 15 min, 30 min, 60 min and 120 min, 7.5 OD600 were harvested in glass tubes, washed with water and the pellet was extracted 3 × 1 ml in water:Ethanol:Diethyl Ether:Pyridine:NH4OH (15:15:5:1:0.018) at 65 °C for 15 min each time. Avanti Polar Lipid MS standards (LM-6002) were added during the first extraction at 62.5 pmol/tube. The extracts were pooled and dried under N2, redissolved in 1 ml Chloroform with bath sonication, 1 ml Butanol was added and phospholipids were hydrolyzed for 30 min at 37 °C after the addition of 200 μL 1 M KOH (in methanol). After hydrolysis, the extract was neutralized by the addition of 200 μL 1 M Acetic Acid (in Methanol). 1 ml Butanol saturated water was added, centrifuged to separate the phases and the upper aqueous layer was removed by aspiration, being careful not to disrupt the precipitate at the interface. This was repeated two more times after which the remaining lower phase was dried under N2. The dried lipid was redissolved in 0.5 ml LC/MS buffer A with bath sonication, spun to pellet insoluble material and the transferred to MS analysis vials.

#### Human neurons

Sphingolipids were extracted by a modified Bligh and Dyer method^49^. Briefly, 1 mL of methanol was added to plates and cells were scraped into glass tubes and 0.5 mL of chloroform containing internal standards (Avanti polar lipids, LM-6002) was added prior to sonication. Following an overnight incubation at 48 °C,150 ul of 1 M KOH (methanol) was added and samples were again briefly sonicated and incubated for 2 h at 37 °C. Samples were neutralized with 12 μL of glacial acetic acid and centrifuged for 5 min to pellet insoluble material. The clarified sample was evaporated under nitrogen and subsequently resuspended 0.4 mL of buffer A:B (80:20), bath sonicated to disperse then centrifuged to clarify and transferred to an HPLC vial for analysis. Typically, 40 μL of each sample was injected for each analysis. The samples were analyzed using an Agilent 1200 Series HPLC coupled to an ABSciex QTRAP 4000 MS.

The column (100 mm ×4.5 uM C18) was pre-equilibrated with buffers A:B (80:20) for 2.8 min at 1 mL/min before injection. After injection, the flow is continued at 80:20 for 0.2 min then ramped to 100% B at 9 min and held at 100% B for 36 min before returning to 80:20 at 50 min. The MS was set to detect ceramides in MRM mode. The MRM parameters were basically as described in Merrill *et al.*^50^. The extraction efficiency, determined by averaging 4 different C12-based sphingolipid internal standards from each sample, was determined to vary by less than 5% for each sample and each sample injection was normalized based on its extraction efficiency. Following LC-ESI-MS profiling, quantitation was performed as described in^51^. Data are expressed as pmol per 1E6 cells. Cell number at time of extraction was determined by counting yoked control cultures plated and treated simultaneously as cultures used for lipid extraction.

### LC/MS Buffers

MS Buffer A: Methanol:H_2_O:formic acid (74:25:1) with 10 mM Ammonium Formate.

MS Buffer B: Methanol:formic acid (99:1) with 10 mM Ammonium Formate.

## Additional Information

**Data availability**: Raw data from microarray experiments can be accessed at NCBI GEO, accession no. GSE40814.

**How to cite this article**: Kennedy, M. A. *et al.* A Signaling Lipid Associated with Alzheimer's Disease Promotes Mitochondrial Dysfunction. *Sci. Rep.* **6**, 19332; doi: 10.1038/srep19332 (2016).

## Supplementary Material

Supplementary Information

Supplementary Dataset 1

Supplementary Dataset 2

Supplementary Dataset 3
