# Supplementary material for: A Signaling Lipid Associated with Alzheimer’s Disease Promotes Mitochondrial Dysfunction
Source: Sci Rep. 2016 Jan 13;6:19332. doi: 10.1038/srep19332 (PMC4725818; doi:10.1038/srep19332)
Supplement: Supplementary Information [file srep19332-s1.pdf]

Supplemental Information

A Signaling Lipid Associated with Alzheimer's Disease Promotes Mitochondrial Dysfunction

Michael A. Kennedy[1], Tia C. Moffat[1], Kenneth Gable[2], Suriakarthiga Ganesan[3], Karolina Niewola-Staszkowska[4], Anne Johnston[5], Corey Nislow[6], Guri Giaever[6], Linda J. Harris[5], Robbie Loewith[4], Vanina Zaremberg[3], Mary-Ellen Harper[1], Teresa Dunn[2], Steffany A.L. Bennett[1,]* and Kristin Baetz[1,*]

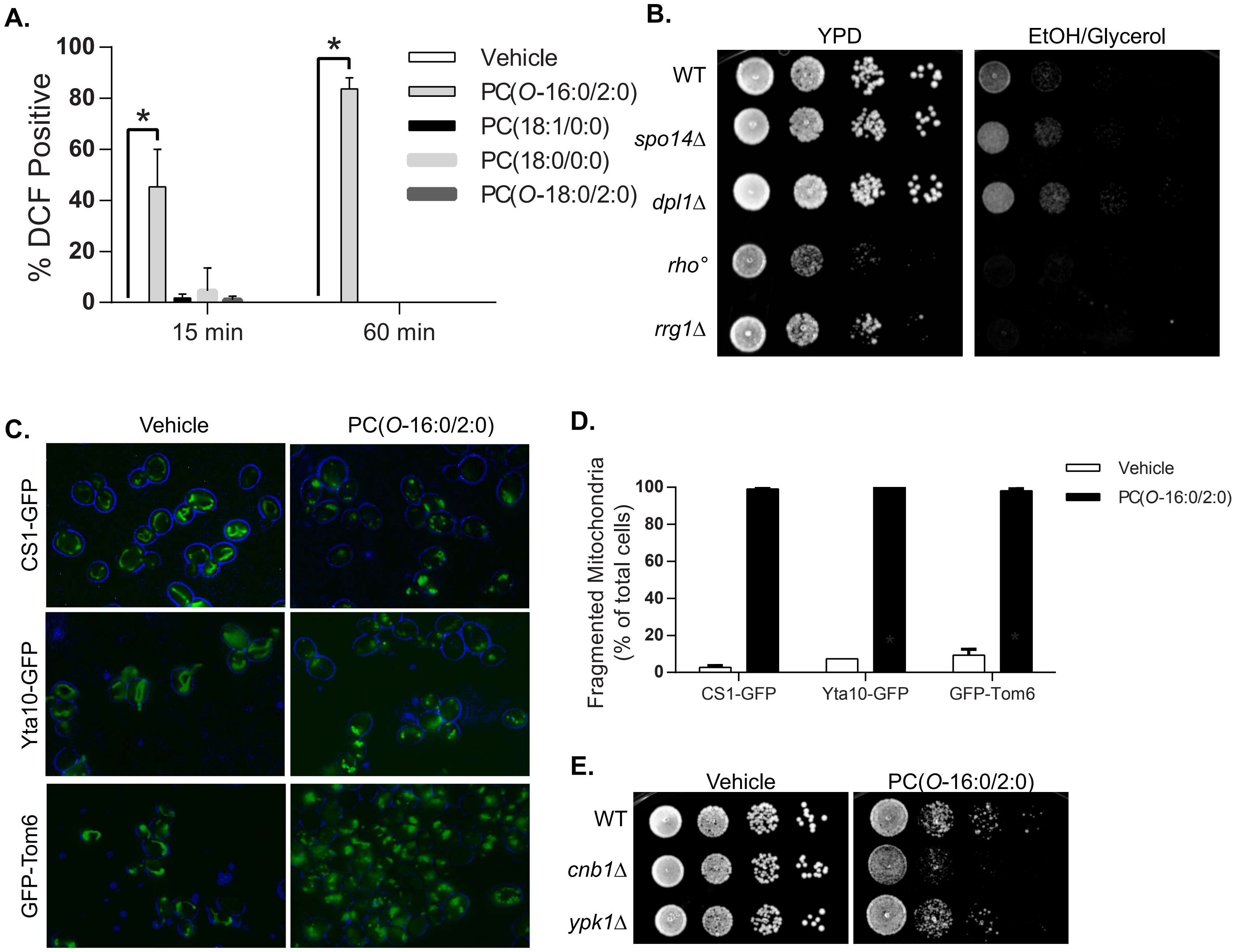

Supplemental Figure 1. **(A)** *H2-DCFDA fluorescence is not increased in cells treated with control lipids. W*ild type (BY4741) cells were grown to mid log in YPD and subsequently treated with Vehicle (0.2% Ethanol) or 20 μM of the indicated lipids for t = 15 or 60 min prior to labelling with H2-DCFDA (10 μM). Quantifications of H2-DCFDA positive cells at the indicated time points from at least three independent experiments where a minimum of 150 cells were counted. ND - not detected. Error bars = SD, (*p<0.01, Kruskal-Wallis, corrected for multiple comparisons). **(B)** *Growth of mitochondrial deficient yeast strains on non-fermentable carbon source.* Wild type (WT, BY4741), *spo14*Δ (YKB3113), *dpl1*Δ (YKB3306), rho° (YKB3925), *rrg1*Δ (YKB3911) strains were tested for the ability to grow on a non-fermentable carbon source (3% v/v ethanol and 3% v/v glycerol). The indicated strains were spotted in 10-fold dilutions and incubated for 2 days at 30°C. **(C)** and **(D)** *PC(O-16:0/2:0) promotes mitochondrial fragmentation.* Wild type (WT, BY4741) cells expressing the indicated mitochondrial markers fused to GFP were incubated with Vehicle or 20 μM PC(O-16:0/2:0) for 1 hour at 30°C and then imaged live using fluorescence microscopy (see Material and Methods). CS1-GFP (matrix), Yta1-GFP (inner membrane), GFP-Tom6 (outer membrane). Mitochondrial fragmentation for each marker was quantified as the percentage of cells displaying a fragmented pattern in the presence of vehicle or PC(*O*-16:0/2:0). n=4, Error bars = SD. *A ypk1*Δ *mutant does not have increased sensitivity to PC(O-16:0/2:0).* Wild type (WT, BY4741), *cnb1*Δ (YKB4240) and *ypk1*Δ (YKB4241) strains were spotted in 10-fold dilutions and incubated for 2 days at 30°C on YPD plates containing vehicle (0.2% EtOH) or PC(*O*-16:0/2:0) (6 μg/ml).

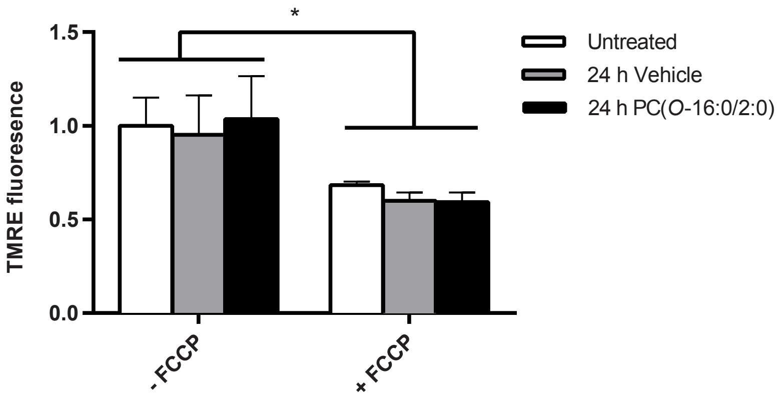

Supplemental Figure 2. *PC(O-16:2/2:0) does not alter ΔΨm in cultured neurons.* hNT neurons were left untreated or treated with vehicle or PC(*O*-16:0/2:0) (1 μM) in serum-free media for 24 hr. Changes in TMRE fluorescence represent changes in intensity averaged over 10 minutes and normalized to μg of protein relative to controls. FCCP was used as a positive control to induce proton leak and decrease mitochondrial membrane potential.

Supplementary Table 1. List of yeast strains and plasmids used.

Name	Genotype	Source
YPH500	***MAT*α** *ade2-101 his3-Δ200 leu2-Δ1 lys2-801 trp1-Δ63 ura3-52*	(*1*)
BY4741	***MATa*** *his3Δ1 leu2Δ0 lys2Δ0 met15Δ0 ura3Δ0*	(*2*)
YKB3911	***MATa*** *his3Δ1 leu2Δ0 lys2Δ0 met15Δ0 ura3Δ0 rrg1Δ::KANMX*	This Study
YKB3925	***MATa*** *his3Δ1 leu2Δ0 lys2Δ0 met15Δ0 ura3Δ0 rho°*	This Study
YKB3271	***MATa*** *his3Δ1 leu2Δ0 lys2Δ0 met15Δ0 ura3Δ0 ypc1Δ::KANMX ydc1Δ::KANMX*	This Study
YKB3273	***MATa*** *his3Δ1 leu2Δ0 lys2Δ0 met15Δ0 ura3Δ0 lcb4Δ::KANMX lcb5Δ::KANMX*	This Study
YKB3305	***MATa*** *his3Δ1 leu2Δ0 lys2Δ0 met15Δ0 ura3Δ0 ysr3Δ::KANMX lcb3Δ::KANMX*	This Study
YKB3306	***MATa*** *his3Δ1 leu2Δ0 lys2Δ0 met15Δ0 ura3Δ0 dpl1Δ::KANMX*	This Study

YKB3927	***MATa** his3Δ1 leu2Δ0 lys2Δ0 met15Δ0 ura3Δ0 dpl1Δ::NATMX lcb4Δ::KANMX lcb5Δ::KANMX*	This Study
CS1-bGFP	pGAL68 plasmid (CEN URA3) for expression of CS1 fused to GFP under the control of a GAL1/10 promoter	(*3*)
Yta10p-bGFP	pGAL68 plasmid (CEN URA3) for expression of Yta10 fused to GFP under the control of a GAL1/10 promoter	(*3*)
bGFP-Tom6p	pGAL68 plasmid (CEN URA3) for expression of Tom6 fused to GFP under the control of a GAL1/10 promoter	(*3*)

References

1. R. S. Sikorski, P. Hieter, A System of Shuttle Vectors and Yeast Host Strains Designed for Efficient Manipulation of DNA in. *Genetics* (1989).

2. C. B. Brachmann, A. Davies, G. J. Cost, E. Caputo, Designer Deletion Strains derived from Saccharomyces cerevisiae S288C : a Useful set of Strains and Plasmids for PCR-mediated Gene Disruption and Other Applications. **132**, 115–132 (1998).

3. K. Okamoto, P. S. Perlman, R. A. Butow, The Sorting of Mitochondrial DNA and Mitochondrial Proteins in Zygotes: Preferential Transmission of Mitochondrial DNA to the Medial Bud. **142**, 613–623 (1998).
